# Distributed Optical Fiber Sensor Applications in Geotechnical Monitoring

**DOI:** 10.3390/s21227514

**Published:** 2021-11-12

**Authors:** Aldo Minardo, Luigi Zeni, Agnese Coscetta, Ester Catalano, Giovanni Zeni, Emilia Damiano, Martina De Cristofaro, Lucio Olivares

**Affiliations:** 1 Department of Engineering, University of Campania “Luigi Vanvitelli”, 80131 Aversa, Italy; luigi.zeni@unicampania.it (L.Z.); agnese.coscetta@unicampania.it (A.C.); ester.catalano@unicampania.it (E.C.); emilia.damiano@unicampania.it (E.D.); martina.decristofaro@unicampania.it (M.D.C.); lucio.olivares@unicampania.it (L.O.); 2 Institute for Electromagnetic Sensing of the Environment, National Research Council, 80124 Naples, Italy; zeni.g@irea.cnr.it

**Keywords:** optical fiber sensing, landslide monitoring, rock slope failures, soil slope failures

## Abstract

We report the experimental application of distributed optical fiber sensors, based on stimulated Brillouin scattering (SBS), to the monitoring of a small-scale granular slope reconstituted in an instrumented flume and subjected to artificial rainfall until failure, and to the monitoring of a volcanic rock slope. The experiments demonstrate the sensors’ ability to reveal the sudden increase in soil strain that foreruns the failure in a debris flow phenomenon, as well as to monitor the fractures in the tuff rocks. This study offers an important perspective on the use of distributed optical fiber sensors in the setting up of early warning systems for landslides in both rock and unconsolidated materials.

## 1. Introduction

**Figure 1:**
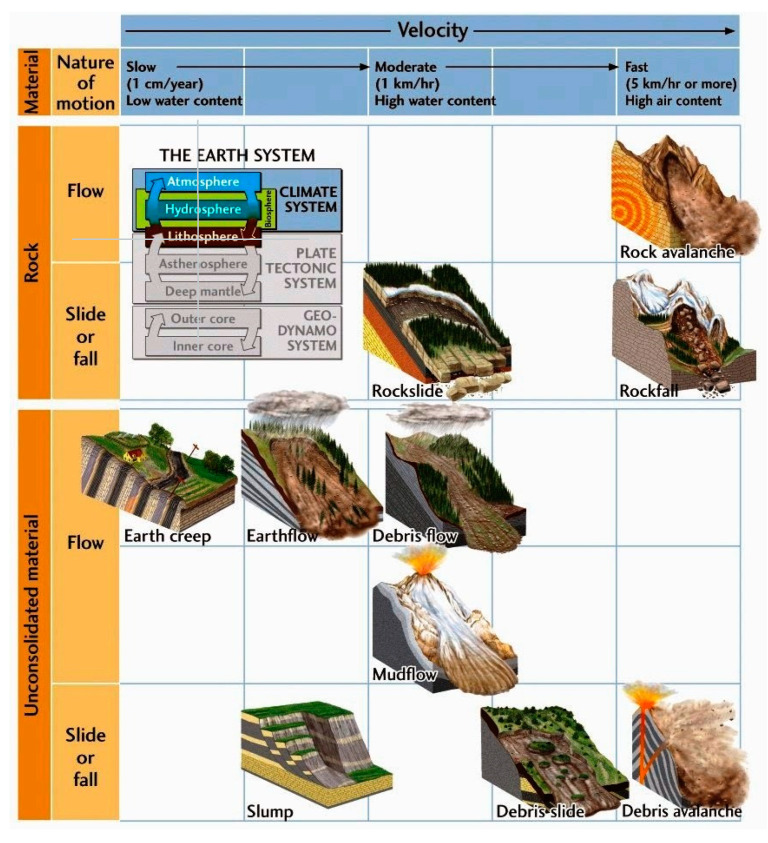
Landslide classification based on the rate and type of movement. From http://www.geologyin.com/2015/02/types-of-wasting-slump-rockslide-debris.html (accessed on 8 November 2021).

Every year, fatalities and economic losses due to hydro-geological phenomena are very high; in this respect, landslides are the third most significant natural disaster worldwide [1]. Although a landslide can be defined as the movement of a mass of rock, debris or earth under gravitational force [2], the type, extension and speed of movements cover a wide range of variation, leading to various classifications. In Europe, on the basis of the EPOCH project [3], the landslides are classified only according to the soil and type of movement, whereas the most diffuse classifications take into the account the speed of these movements (Figure 1), which is one of the most variable characteristics. A landslide can travel at a rate ranging from few centimeters per year, as in the case of earthflows or deep-seated complex phenomena, to a few km per hour, as in the case of rock-fall or debris avalanches, which allow no time to alert populations and cause huge damages to both constructions and humans.

To reduce the risk of landslide, one of the main strategies is the use of early-warning systems (EWS) based on effective monitoring: the triggering causes, if clearly recognized, or their effects, such as slope displacement and acceleration, can be monitored. In some cases, such as when a landslide is so large that it cannot be stabilized, the adoption of EWS can be the only practical solution [4,5]. However, due to the great variability of the phenomena under observation, no universal monitoring set-up can be used. In the case of rapid and very rapid slope movements, the detection of superficial ground displacement in real time represents one of the most effective tools for slope failure prediction [6,7]. Indeed, continuous surveillance allows the immediate detection of movements, which can be crucial to making timely decisions about safety and to the adoption of subsequent early warning actions. Moreover, the availability of remote, effective monitoring systems also deepens the understanding of the landslide kinematics.

Although conventional geodetic and geotechnical instrumentations, such as total stations, GPS receivers, inclinometers, or extensometers, are very accurate and reliable, they provide measurements only over a few points and not over the whole unstable area. Furthermore, they are not effective at monitoring either very rapid landslides, which require high time resolution monitoring, or the mass movement of the first detachment, where the location of the area prone to landslide is not recognizable a priori. Additionally, distributed optical fiber sensors can be applied, as they allow the monitoring of large areas with high accuracy and time frequency at a low cost. Slope movements can be detected and localized by properly anchoring the fiber to the soil either directly or through the use of geogrids/geotextiles [8,9,10,11].

In this paper, we report two examples of the use of distributed optical fiber sensors as strain monitoring systems for two different types of slope movements, highlighting the versatility of this tool. In one example, the sensors monitor and analyze the deformation of a small-scale slope, in which a debris flow is induced, while in the other example they are applied to monitor the movements along cracks in a rocky slope made of volcanic tuff. The experimental results show that the sensing fiber can detect soil movements well before the occurrence of slope failure, as well as the formation, the evolution, and the location of fractures on tuff rocks.

In the following sections, we first describe the methodology. Subsequently, the experimental measurements are reported. Conclusions follow at the end of the paper.

## 2. Methodology

Optical fiber sensors are well suited to geotechnical monitoring applications thanks to their advantages over other technologies, such as their tolerance of aggressive environments, their ease of integration into tight areas within structural components and their multiplexing capability [12]. Fiber Bragg grating (FBG) sensors have been considered as reliable sensors for health monitoring of structural and geotechnical projects [13]. However, they only provide strain sensing at discrete points along the fiber. By contrast, distributed optical fiber sensors provide spatially continuous sensing over the entire length of the fiber; therefore, they are more effective than FBGs, especially when monitoring over a large area is required. Distributed optical fiber sensors based on stimulated Brillouin scattering (SBS), in particular, offer the opportunity to perform temperature and/or strain measurements in optical fibers over long distances (up to several tens of km) and with high spatial resolution (one meter or less). In these sensors, a probe wave interacts with a frequency-shifted pump wave, counter-propagating along the fiber. When the frequency shift between the two waves falls within the so-called Brillouin gain spectrum (BGS), a power transfer occurs between the two waves, leading to a frequency-dependent amplification of the probe. The Brillouin frequency shift (BFS) represents the frequency offset for which the maximum probe amplification occurs. Any deviation of the BFS from the reference measurement is a sign of a strain and/or temperature change. The Brillouin optical time-domain analysis (BOTDA) and the Brillouin optical frequency-domain analysis (BOFDA) techniques provide a spatially resolved measurement of the BFS along the fiber; however, they differ in the type of modulation impressed on the pump wave: in the BOTDA method, a pulsed pump wave is employed [14], while the BOFDA sensor requires the use of a sinusoidally-modulated pump beam [15]. The different modulation format leads to different characteristics of the two sensing methods: BOTDA sensors are usually limited to a meter-scale spatial resolution, due to the time required to trigger the SBS mechanism. On the other hand, BOFDA sensors are capable of much better spatial resolutions (in the cm or even mm range), thanks to the pre-activation of the acoustic wave involved in the SBS phenomenon. From the point of view of the acquisition time, BOFDA measurements are usually slower because of the necessity to perform time-consuming frequency sweeps using a vector network analyzer. For sensing fibers that are not excessively long (less than 1 km), BOTDA measurements are usually completed in fractions of seconds, while BOFDA measurement times are in the minutes range.

**Figure 2:**
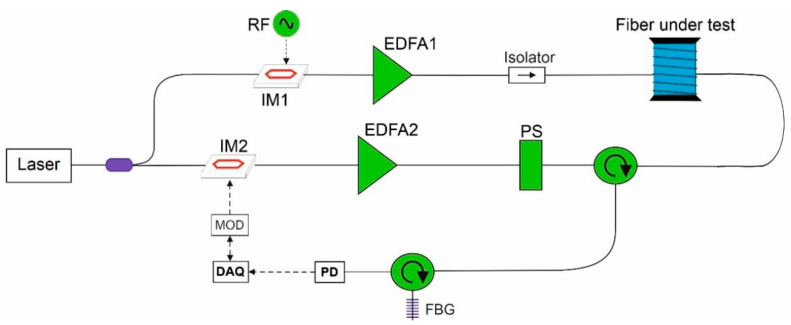
Experimental setup. EDFA: erbium-doped fiber amplifier; PS: polarization switch; IM: intensity modulator; FBG: fiber Bragg grating; PD: photoreceiver; DAQ: data acquisition; MOD: modulating signal generator. The solid lines indicate the fiber-optic paths, while the dashed lines indicate the electrical paths.

The setup shown in Figure 2 implements a generic scheme for BOTDA or BOFDA measurements. Light from a 1550-nm laser (CoBrite DX1, ID Photonics, Neubiberg, Germany) is split by a 50/50 optical coupler. In the lower branch, the light is modulated through an electro-optic intensity modulator (IM2), in order to impress either a pulsed or a sine-wave modulation, depending on the type of measurements to be made (BOTDA or BOFDA, respectively). The consequent pump signal is amplified through an erbium-doped fiber amplifier (EDFA2), polarization-switched and, finally, sent to one end of the fiber under test (FUT) through an optical circulator. The polarization switch (PS) rotates by 90°, alternately, the state of polarization of the pump wave, in order to implement a polarization-diversity scheme [9]. The upper branch shows the generation of the probe wave through a double-sideband suppressed-carrier modulation. In practice, the intensity modulator IM1 is biased at the minimum of its transmission, so that the applied radiofrequency (RF) signal (as provided by the microwave generator SynthHD, Windfreak Technologies, Port Richey, FL, USA), generates two sidebands in the probe wave spectrum, with the distance of each one from the laser frequency corresponding to the RF frequency. The probe wave is then amplified through the EDFA1 and injected into the other end of the FUT. The backscattered light is sent to a narrowband (~0.04 nm) fiber Bragg grating (FBG), which selects the sideband at the lower frequency (Stokes component) and, at the same time, filters out the amplified spontaneous emission noise of EDFA1. Finally, the light is converted to an electrical signal through a high-speed photoreceiver (PD) (1544-B, Newport Corporation, Irvine, CA, USA) connected to a data acquisition system (DAQ) consisting of a digitizer (FMC104, Abaco Systems, Huntsville, AL, USA), or a vector network analyzer (M9374A, Keysight, Santa Rosa, CA, USA) for BOTDA or BOFDA measurements, respectively.

## 3. Experimental Tests in an Instrumented Flume

The distributed optical fiber sensor was used in experimental tests aimed at investigating the deformation process up to failure of small-scale volcanic slopes subjected to artificial rainfall [16]. The soil used in the experiments was taken from the Cervinara site (North-East of Naples), an area affected by a catastrophic debris avalanche in 1999. The soil, comprising granular cohesionless volcanic sand [17], was laid down by placing 1 cm thick layers, which were gently tamped while wetted, reaching a total thickness of 10 cm. The slope length and width were 110 cm and 50 cm, respectively. Due to the limited size of the test rig, it was essential to perform distributed strain measurements with high (cm scale) spatial resolution. Therefore, the BOFDA method was chosen for this test, ensuring a spatial resolution of 5 cm at the expense of a time resolution of about three minutes.

In the described experiment, the imposed slope angle was 35°, significantly lower than the friction angle of the soil equal to 38° and, in order to favor a mechanism of progressive failure, the 35 cm long uppermost part of the slope was 5° steeper than the remaining part.

**Figure 3:**
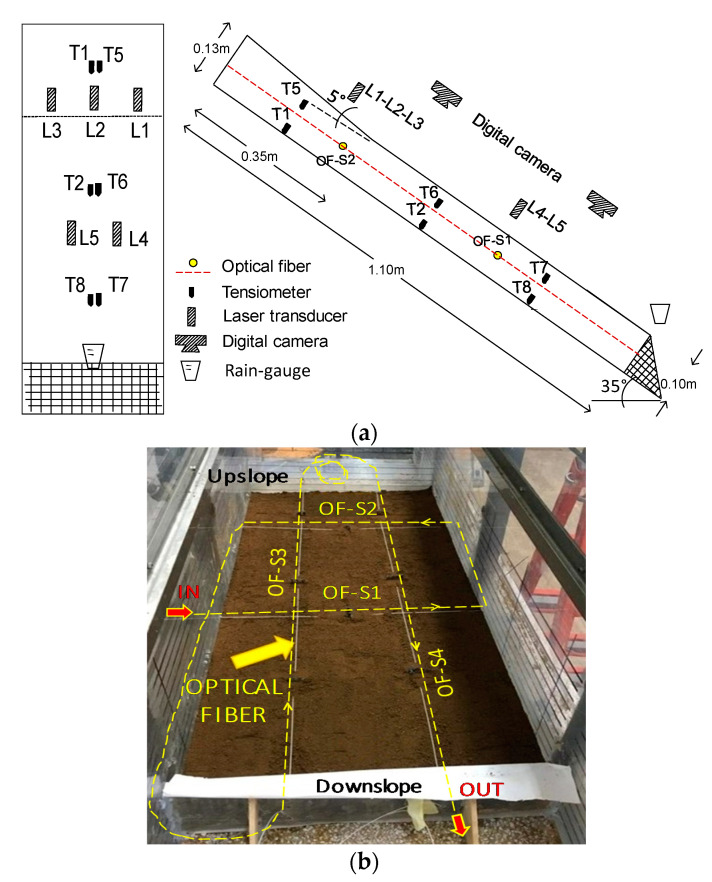
Slope with location of the sensors: schematic plan-view and cross section (**a**), and fiber installation during the reconstitution of the slope (**b**).

A 0.9 mm tight-buffered optical fiber was installed during the formation of the slope at a depth of 5 cm (i.e., at mid-depth), in order to measure the soil strain field (see Figure 3b). The optical fiber wire crossed the slope along two transversal sections (OF-S1 and S2 in Figure 3) and two longitudinal sections (OF-S3 and S4 in Figure 3). The fiber strands inside the slope were slightly pretensioned and fixed at the plexiglass walls of the flume by glue. To enhance the stress transfer between the sensing cable and the surrounding soil, small anchors were produced by using pieces of geonet located along the optical fiber each 25 cm.

During the test, other devices were used to monitor the slope’s behavior: six miniaturized tensiometers installed at mid-depth (5 cm of depth) and at the bottom (10 cm of depth) of the slope at three different points for suction measurements (Figure 3a), and five laser sensors installed above the ground surface for monitoring soil settlements. In addition, two digital photo cameras were used to retrieve, through Particle Image Velocimetry (PIV), the displacement and velocity field of the ground surface, integrating the monitoring system. The two cameras, installed at a height of 1 m from the slope surface, allowed framing an area 50 cm wide and 100 cm long. A schematic cross-section and plan-view of the instrumented slope is reported in Figure 3.

To induce slope failure, an artificial rainfall intensity of 100 mm/h was reproduced above the ground surface. The soil was initially unsaturated, and this conferred on it a shear resistance higher than when saturated. The progressive wetting of the slope induces an increase in the soil unit weight and a decrease in the soil strength, up to the opening of tension cracks and the triggering of slope failure which, in this loose metastable material, evolves into a very rapid debris flow.

**Figure 4:**
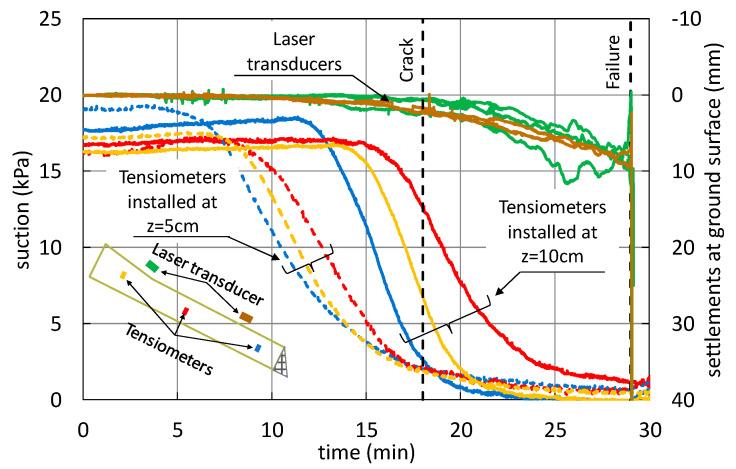
Suction and settlement trends during the infiltration test. Suction values refer to tensiomenters installed at z = 10 cm (solid lines) or z = 5 cm (dashed lines).

Figure 4 shows the effects of rainfall on soil suction (left axis of Figure 4) and deformation of the slope surface in terms of settlements (right axis of Figure 4). Note that the different colors of the lines in the plot refer to different positions of the sensors, as illustrated by the inset in the left bottom corner of the figure. Suction progressively reduces up to a nihil value (t ≈ 25 min), indicating soil saturation, as measured by tensiometers installed at the bottom of the slope (z = 10 cm).

During the wetting process, cracks began to appear on the ground surface (t ≈ 18 min) in an area just below the slope change, slightly below the position of the OF-S2 (Figure 3b). At this point, the laser sensor transducers recorded settlements of about 5 mm all over the slope (t ≈ 25 min) (Figure 4), which indicated that both volumetric and shear strains were occurring. Moreover, the soil suction recorded at the bottom of the slope (tensiometers installed at z = 10 cm in Figure 4) became lower than the suction recorded at mid-height (tensiometer at z = 5 cm in Figure 4), indicating that soil saturation was occurring, with the possible formation of a water table arising from the bottom of the slope. Indeed, the progressive soil saturation accompanied by volumetric and shear strains is the mechanical process leading to slope failure [18,19]. Afterwards, the settlements increased significantly and in a less uniform manner throughout the slope, up to failure, which occurred 29 min after the beginning of the rainfall.

**Figure 5:**
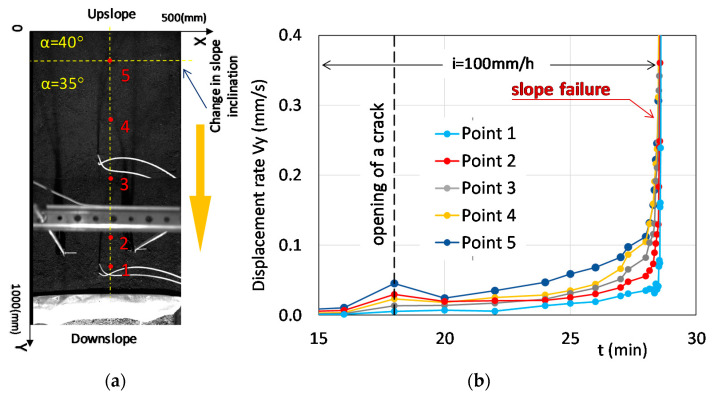
Monitored points at ground surface (**a**) and displacement rate trends retrieved by PIV technique (**b**).

Slope deformation was also analyzed by camera recording and by optical fiber sensors. A progressive slope deformation was revealed by the displacement rates along the slope section A-A,’ illustrated in Figure 5 and retrieved by PIV: the uppermost part of the slope, where the slope inclination was higher (point 5 in Figure 5), started to move earlier with a higher velocity than the lowermost part of the slope and the deformation process remained characterized by a higher speed in the upper part of the slope during the entire experiment. As the toe of the slope was fixed, the displacement rate was clearly characterized by the smallest values at point 1.

**Figure 6:**
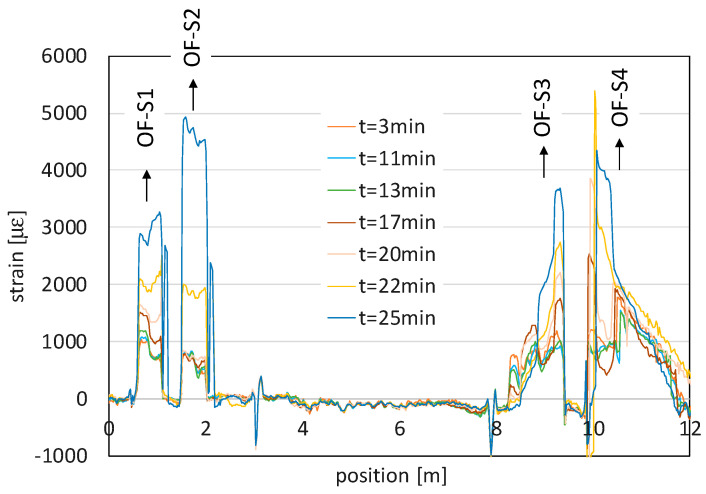
Strain trends measured by the optical fiber sensor along its entire length (sections OF S1-S4 indicate the strands embedded into the slope).

This was also confirmed by the optical fiber measurements, illustrated in Figure 6, in terms of strain trends along the entire length of the cable: the strands of fiber embedded into the slope are clearly distinguishable, since they are the only portions subject to strain. The strain values were extracted by first subtracting each BFS profile to the one acquired at the beginning of the test (i.e., at t = 0 min) and then converting the BFS changes in the strain values by adopting a conventional transduction factor of 0.05 MHz/µε. The sensor followed the progressive soil deformation, which gradually increased with time everywhere in the slope. It also distinguished the different behavior of the upper part of the slope (OF-S2 and abscissa around 9.3 m and 10.2 m of OF-S3 and OF-S4 in Figure 6), characterized by strain values higher than those measured in the middle and lower part of the slope.

Looking at the part of the fiber sensor installed at the toe of the slope, corresponding to the abscissa around 8.2 and 11.3 in Figure 6, it is clear that although the fiber revealed very small values of strains, the sensor was not able to distinguish the compressive strains expected at the toe of the slope. This was probably due to the imperfect stress transfer between the fiber and the surrounding soil.

**Figure 7:**
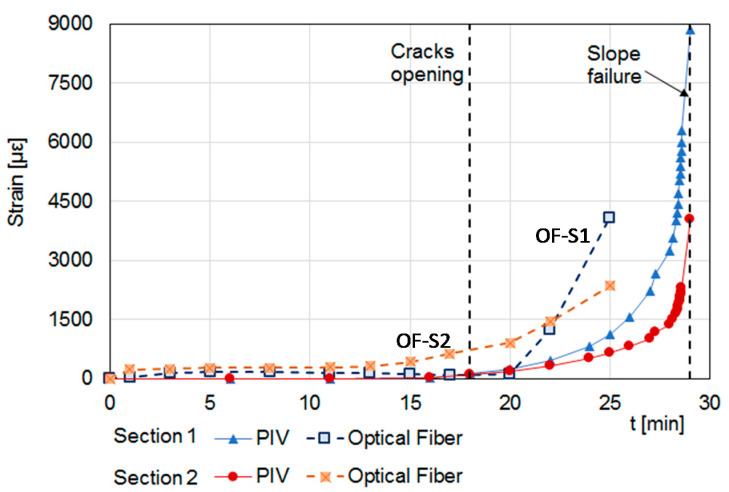
Strains measured by the transverse optical fiber strands and by digital camera at location of optical fiber OF-S1 (45 cm from downslope) and OF-S2 (30 cm from upslope).

Finally, Figure 7 shows a comparison between the strains measured by the fiber sections disposed crosswise (transverse) in the flume (sections OF-S1 and OF-S2) and those retrieved by the digital camera using the PIV technique. The strain measured by the two types of techniques were in good agreement during the first part of the experiment while, after the opening of tension crack, only the optical fiber sensor revealed that the strain greatly increased from about 10 min before the slope failure. Moreover, the fiber strand OF-S2, located close to the area where the tension cracks occurred, was able to detect a soil strain higher than that revealed by the PIV at the same position (Section 2 in Figure 7) and at an early time (about 4 min): one minute before the opening of cracks on ground surface the OF-S2 recorded a soil strain of about 630 με, whereas that revealed by the PIV was less than 100 με. This indicates that the optical fiber sensor can be effectively used, not only to evaluate the slope strain field during a rainwater infiltration process, but also to detect in time the precursor signals of an incoming failure.

## 4. Experimental Tests on a Coastal Cliff

**Figure 8:**
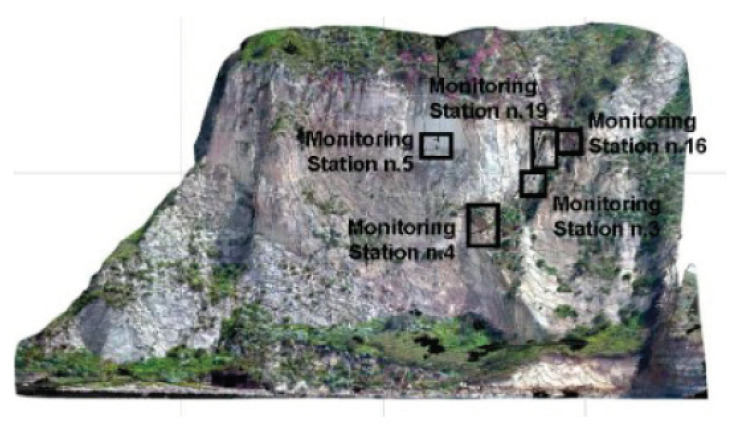
Frontal view of Coroglio cliff. The monitoring stations n. 3 and n. 19 were chosen for the distributed optical fiber sensor. The other monitoring stations were served by conventional crackmeters (Ref. [22]).

**Figure 9:**
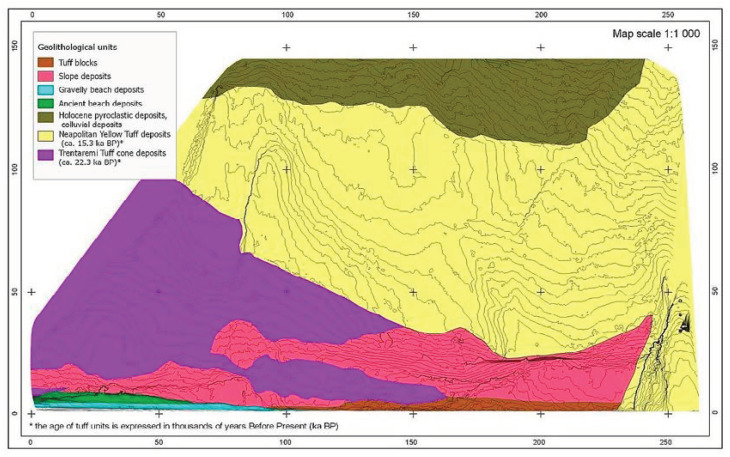
Geological map of Coroglio cliff (Ref. [20]).

The distributed optical fiber sensor was also used to monitor the Coroglio tuff cliff for three years (May 2015–May 2018). The coastal cliff of Coroglio (Posillipo hill, Naples, Italy), in the Campi Flegrei coastal area, is representative of tuffaceous coastal cliffs in densely populated areas; it was chosen due to its slope morphology, its exposure to marine erosion (due to the wave action, wind abrasion, thermal excursions, etc.) and its type of geological substratum and anthropic activities [20,21]. The Coroglio sea cliff is 140 m high and 250 m wide (see Figure 8). The geological map is shown in Figure 9. The upper part of the cliff features slope angles ranging from 35° to 45° and is characterized by loose Holocene pyroclastic deposits about 30 m thick. The median sector of the cliff features an almost vertical slope, and is formed by two tuffaceous units, separated by an unconformity. Of these units, the upper one is made up of Neapolitan Yellow Tuff, a lithified ignimbritic deposit formed by alternating coarse-grained matrix-supported breccia, thin-laminated lapilli beds and massive ash layers, while the lower unit is represented by the oldest tuff cone of Trentaremi, consisting of coarse-grained pumice and lapilli beds. At the base of the cliff slope, talus breccia and beach deposit also occur. A complex system of structural discontinuities and fractures characterizes the volcaniclastic succession of the Coroglio cliff [20], with mostly steep and planar fractures of highly variable density.

**Figure 10:**
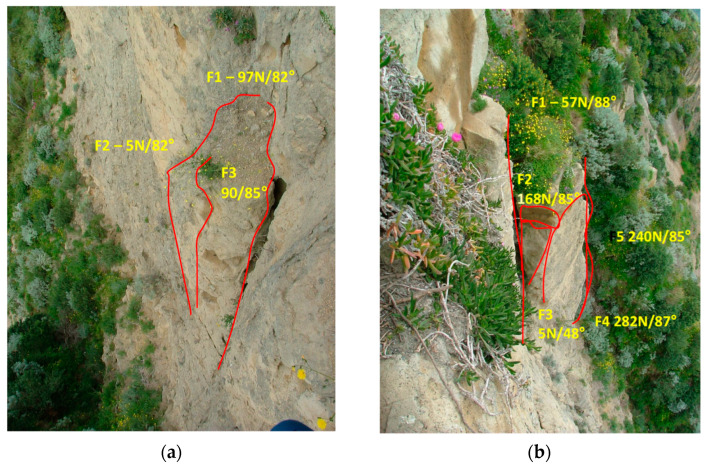
Tuff blocks monitored by the distributed optical fiber sensor: block n. 3 (**a**) and n. 19 (**b**).

A 30 m long strain sensing cable was fixed to a number of nails across the tuff blocks located in the upper part of the cliff. In particular, two deployment zones were selected for optical fiber measurements (block n. 3 and block n. 19 in Figure 8). Block n. 3 is about 4 m high, 2 m wide, 1.2 m thick, being isolated behind fracture F1, and laterally from fracture F2 (Figure 10a). The F1 fracture is very open and contributes to the formation of a rocky wedge intersecting with the other present fractures. Block n. 19 is about 5 m high, 2 m wide, and 1.5 m thick; it is isolated behind fracture F1, which can cause overturning (Figure 10b). There are also lateral fractures (F3 and F5). The block appears thinned to the foot.

**Figure 11:**
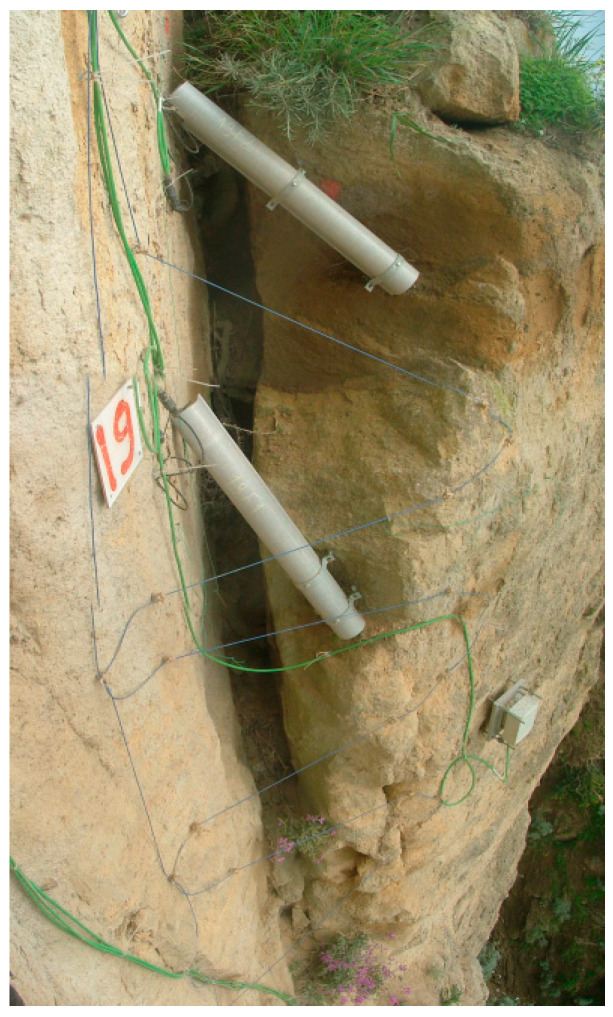
Installation of the sensing cable in the tuff block n. 19.

The sensing cable was a BRUsens V1 cable (Brugg Kabel AG, Brugg, Switzerland) characterized by a 2.8-mm Ethylene propylene rubber (EPR) outer sheet. The installation of the sensing cable was performed by anchoring the fiber in hairpin curves around the fracture to be monitored (see Figure 11). The anchors were produced by fixing 6 mm closing screws in the tuff, sealed with bicomponent epoxy resin inside drilled holes. The fiber was then fixed to the anchors with plastic clamps until it reached a sufficient tension; next, the cable/screw anchor point was definitively sealed with bicomponent epoxy resin (2-Ton Epoxy, ITW Performance Polymers, Danvers, MA, USA). Block n. 3 was monitored with three optical fiber strands across the fracture, while block n. 19 was provided with five optical fiber strands.

**Figure 12:**
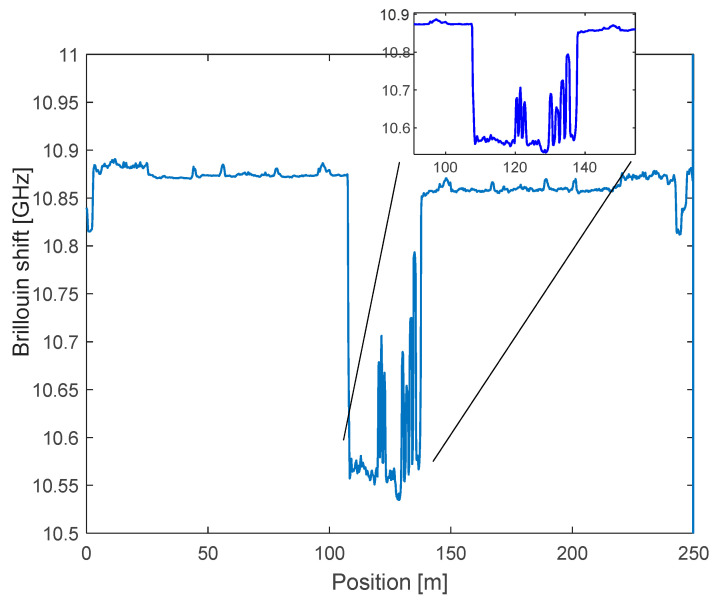
BFS profile measured along the whole fiber after installation. The inset shows a zoom of the sensing region.

Figure 12 reports the Brillouin frequency shift (BFS) profile measured after the installation of the cable, as acquired at the spatial resolution of 50 cm. For these measurements, the less stringent requirement in terms of spatial resolution led us to the choice of a time-domain configuration (BOTDA). The figure highlights the composition of the cable: the first 105 m, characterized by a BFS of ~10.87 GHz, were only aimed at reaching the site of monitoring from the top of the ridge, where the interrogation unit was positioned; the following 33 m (from 105 m to 138 m), characterized by a BFS of ~10.59 GHz, comprised the V1 sensing cable; the final 105 m (from 138 m to 243 m) were required to close the loop configuration. The two 105 m fiber lengths used to carry the signal into the monitored region were composed of two fibers enclosed in a single, armored cable. Notably, the difference in the BFS between the V1 sensing cable and the armored cable should simply be attributed to a different concentration of dopant in the core.

From the inset of Figure 12, the three peaks associated with tuff block n. 3 and the five peaks associated to tuff block n. 19 can be easily distinguished thanks to the pre-tension applied during installation.

**Figure 13:**
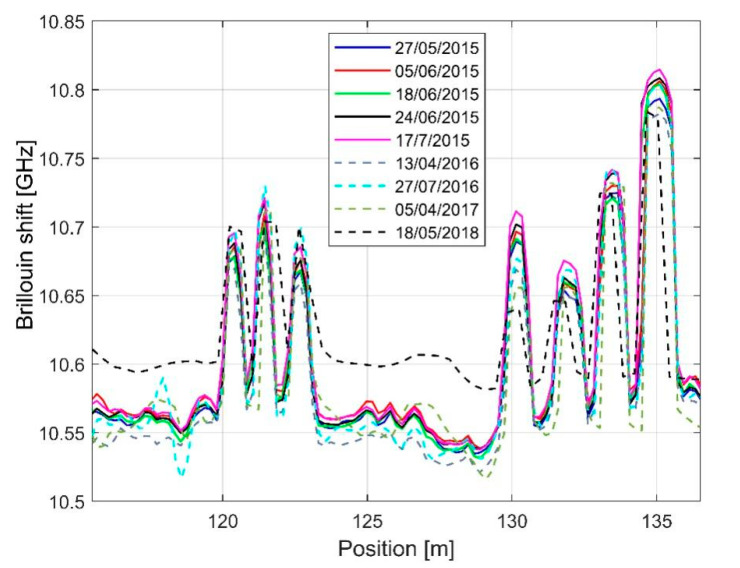
BFS profiles measured along the whole fiber during the monitoring campaign.

In Figure 13, we report the overall results of the monitoring campaign. In particular, the figure shows the BFS distribution in the monitored region, i.e., the points at which the peaks associated to block n. 3 and block n. 19. The figure also reveals that the various curves shifted vertically, which can be attributed to the dependence of the BFS on the temperature (approximatively 1 MHz change of the BFS per °C). The effect of the temperature on the acquired measurements was compensated for by using free sections of the same sensing fiber cable. In fact, strands of the sensing cable were not anchored across the blocks but deployed in near proximity and free standing, so that they only sensed the temperature changes acting as temperature probes.

**Figure 14:**
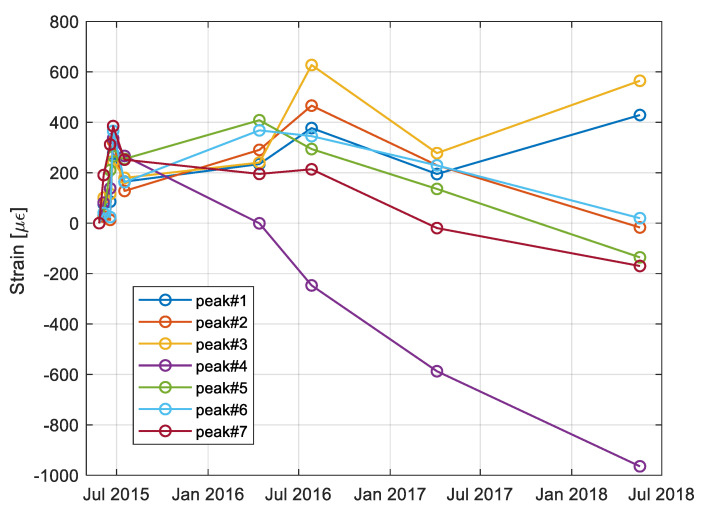
Strain variation of the peaks during the measuring campaign.

By comparing the various measurements taken during the survey, it was observed that the maximum BFS changes were in the order of 30 MHz, which correspond to about 600 με. It can be recalled, as a reference, that a strain of 600 με over a length of 50 cm of stretched fiber corresponds to a longitudinal displacement of 300 μm. The strain variations of the peaks during the monitoring campaign are illustrated in Figure 14.

The obtained results show that the sensing optical fiber system is able to follow the deformations of the blocks during the entire monitoring period, even though the small values recorded were mostly related to periodic, thermal dilations of the rock. In the specific case of block n. 19, the trend of peak 4 was also compatible with the progressive closure of the fracture. This is also confirmed by independent measurements performed with traditional crackmeters and reported in Ref. [23], where long-term (from 2014 to 2018) monitoring results from the same tuff cliff are reported. In particular, block n. 19 exhibited an alternating behavior (due to meteorological conditions), but with a clear prevalence of negative deformations, corresponding to a progressive closure of the fracture.

## 5. Conclusions

Rapid rockfall and debris flows in volcanic rocks and soils, which are widespread around the city of Naples, Italy, affect densely urbanized areas, with a potentially significant impact on the population. To reduce the associated landslide risk, the setup of innovative sensors for the real-time detection of slope movements, able to overcome the limitations of the current systems, is mandatory.

In this paper, we explored the possibility of using optical fibers as soil strain sensors for the setting up of an effective monitoring system for both types of slope movements. In the first case, two fractured tuff blocks at the Coroglio cliff were instrumented and the results of the three-year-long monitoring activities show that the sensing fiber system is able to follow both the thermal dilations of the rock and the progressive deformation of the fracture. In the second case, a small-scale slope reconstituted in volcanic sand was subjected to rainfall until it failed. In this case, a complex monitoring system was installed that allows the testing of the effectiveness of the optical fibers. The results highlighted that the optical fibers detect the progressive deformation of the slope earlier than the other sensors and are also able to distinguish between the upslope and downslope deformation fields.

As regards the distributed sensing technology, we have shown that both time-domain (BOTDA) and frequency-domain (BOFDA) methods can usually be applied in the field of geotechnical monitoring, with the first method being more suitable in long-range, real field environments, and the second method being preferred for small-scale, high-resolution laboratory experiments.

In conclusion, the experimental tests confirm the great potential of distributed optical fiber sensors for monitoring and analyzing deformation in both cohesionless soil covers and rock slopes. The experiments demonstrate that the early detection of crack opening and of soil slope failure can be obtained and the development of early warning systems is an attainable goal of research.

## Data Availability

Data underlying the results presented in this paper are not publicly available at this time but may be obtained from the authors upon reasonable request.
